# Spectral and time-resolved photoluminescence of human platelets doped with platinum nanoparticles

**DOI:** 10.1371/journal.pone.0256621

**Published:** 2021-09-01

**Authors:** Karina Matveeva, Andrey Zyubin, Elizaveta Demishkevich, Vladimir Rafalskiy, Ekaterina Moiseeva, Igor Kon, Anna Kundalevich, Viktoria Butova, Ilia Samusev

**Affiliations:** REC «Fundamental and Applied Photonics, Nanophotonics», Immanuel Kant Baltic Federal University, Kaliningrad, Kaliningrad region, Russia; Universiti Brunei Darussalam, BRUNEI DARUSSALAM

## Abstract

This paper describes a detailed study of spectral and time-resolved photoprocesses in human platelets and their complexes with platinum (Pt) nanoparticles (NPs). Fluorescence, quantum yield, and platelet amino acid lifetime changes in the presence and without femtosecond ablated platinum NPs have been studied. Fluorescence spectroscopy analysis of main fluorescent amino acids and their residues (tyrosine (Tyr), tryptophan (Trp), and phenylalanine (Phe)) belonging to the platelet membrane have been performed. The possibility of energy transfer between Pt NPs and the platelet membrane has been revealed. Förster Resonance Energy Transfer (FRET) model was used to perform the quantitative evaluation of energy transfer parameters. The prospects of Pt NPs usage deals with quenching-based sensing for pathology’s based on platelet conformations as cardiovascular diseases have been demonstrated.

## Introduction

According to the World Health Organization (WHO) reports, cardiovascular deceases have been remaining the leading cause of death at the global level for the last two decades. The number of deaths has been increased up to nearly 9 million in 2019 [1]. The COVID-19 pandemic has resulted in cardiovascular decease (CVD) increase, which caused deaths in many countries [2–4]. Thrombus formation issues play an important role in the occurrence, diagnosis and treatment of cardiovascular diseases. Platelets are responsible for thrombus formation processes and have been intensively studied worldwide. These blood elements are non-nuclear and their main physiological functions include prevention of blood loss, thrombus formation, secretion and participation in hemostasis. In addition to the above functions, platelets are assigned an important role in pathophysiological processes such as antimicrobial defense, atherosclerosis, rheumatoid arthritis and tumor metastasis [5–10]. Although at present time there is sufficient literature data on the occurrence, development, structure and main functions of platelets [11–14], investigation of the spectral properties of the platelets/single platelet remains a challenging task today. As the platelet activation is a key moment in the pathogenesis of cardiovascular complications, therefore, the inhibition of the platelet specific for antiplatelet therapy receptors as cyclooxygenase [15] or P2Y12 [16] is the most important task in the course of treatment and prevention of CVD complications. Inhibition of platelet aggregation is currently the main therapeutic approach in CVD treatment and prevention. Recently, more and more attention is paid to the issue of variability of response to antiplatelet therapy [17]. The understanding the platelet structure and its spectral response to the antiplatelet therapy is the key to personalized medicine today.

Metal NPs are widely used for blood components investigation and can be applied in theranostics, visualization, biosensorics and photothermal therapy etc. due to their unique properties [18–21]. Localized surface plasmon resonances (LSPR), induced near the metal NPs is considered to be a powerful tool in plasmon-enhanced steady-state [22] and time-resolved [23,24] spectroscopy techniques applied for organic and inorganic media investigation. Plasmonic materials such as silver, gold and copper are well studied and these metals are mostly used for platelet investigations in the visible and near infrared (IR) range [25,26]. A wide range of plasmonic devices, such as nanoantennas [27], gold/gold-shell nanorods [28], and NPs, deposited on different surfaces [29] can be created and successfully used for sensory purposes. Platinum, rhodium, bismuth and aluminum can be used for study in the near UV range. Noble metals such as platinum and rhodium are quite promising in biophysics due to their chemical inertness. They are also not susceptible to oxidation and the formation of an oxide film on the NP surface (unlike aluminum), which can significantly affect their sensory properties [30]. The platelet fluorescence is caused by aromatic amino acid complex consisting of Tyr, Trp, and Phe, which are components of platelet membrane [31] and platelet receptors [32]. Since the absorption spectra of these amino acids belong to ultraviolet (UV) region and overlap with the plasmon absorption spectrum of platinum NPs in the same spectral region, it makes possible to observe and investigate photophysical processes by means of FRET analysis of energy transfer [33] constants between NPs and platelets. Despite FRET-based techniques is widely used for biophysical research in visible and IR range [34], its application for UV plasmonics using Pt NPs is challenging and can expand the field of biophysical research. Platelet membrane, its receptors structure, platelet proteins conformations can be evaluated by means of FRET and Tyr, Tpr, Phe content.

Our paper performs new results for spectral and time-resolved investigations for plasmon depended photoprocesses in complexes of platelets and platinum NPs. Fluorescence, quantum yield and platelet amino acid lifetime changes in presence and without femtosecond ablated platinum NPs have been studied. The possibility of energy transfer between Pt NPs and platelet proteins has been demonstrated. The quantitative evaluation of energy transfer parameters based on FRET quenching has been performed. The achieved results can be used for biophysical FRET-based sensors dealing with UV range.

## Materials and methods

### FDTD simulation

**Fig 1:**
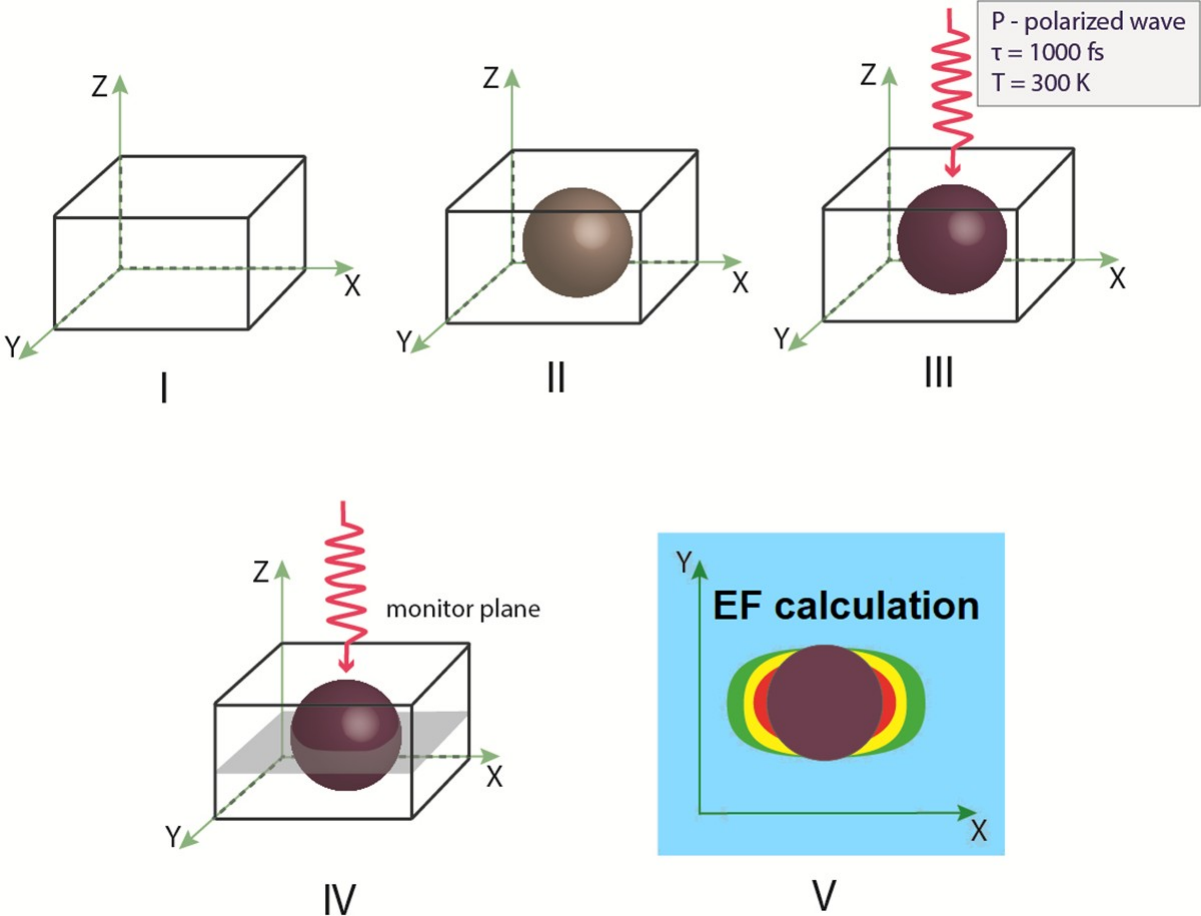
The schematic diagram of FDTD simulation process.

**Fig 2:**
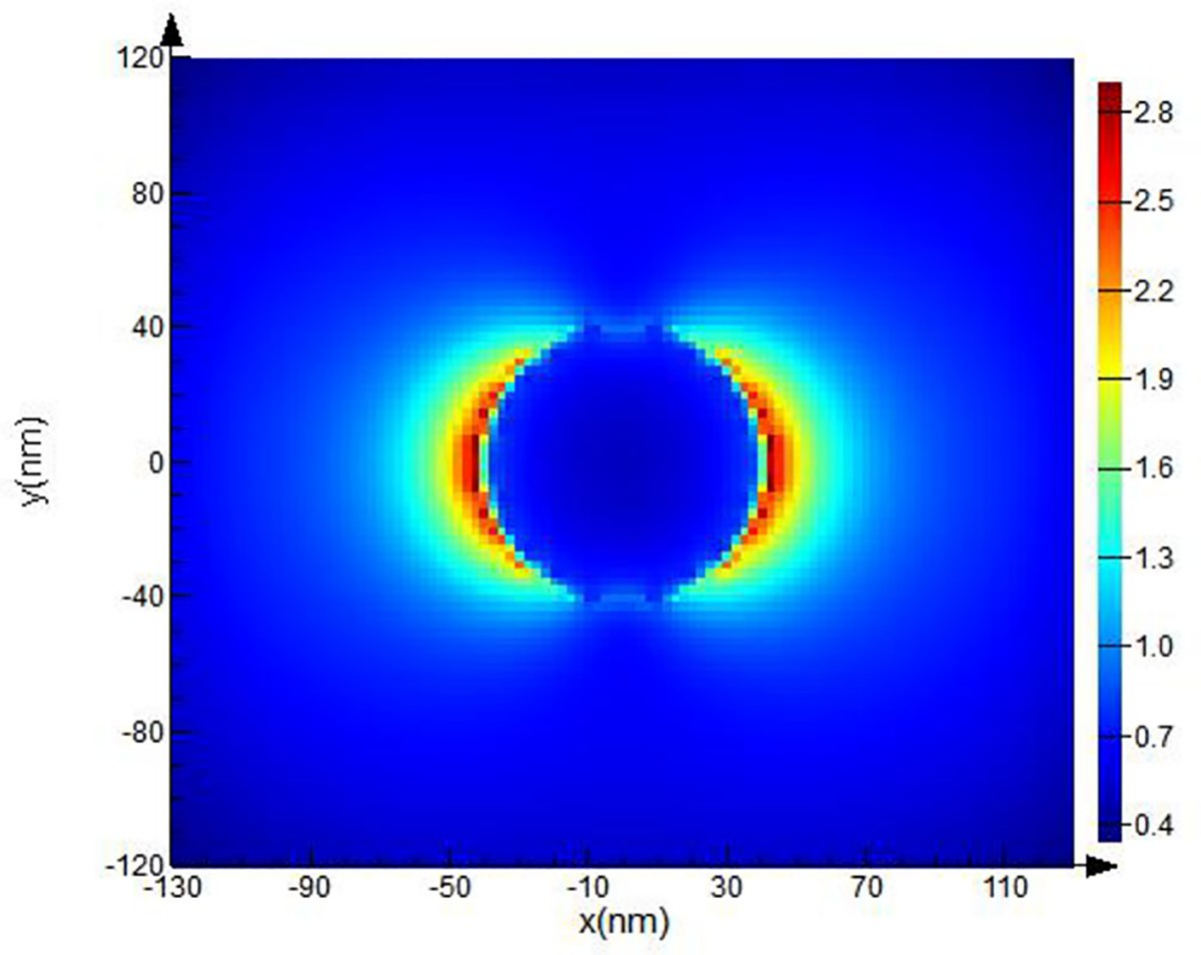
FDTD model of Pt NPs with 40 nm radii for λ = 251 nm excitation wavelength.

**Fig 3:**
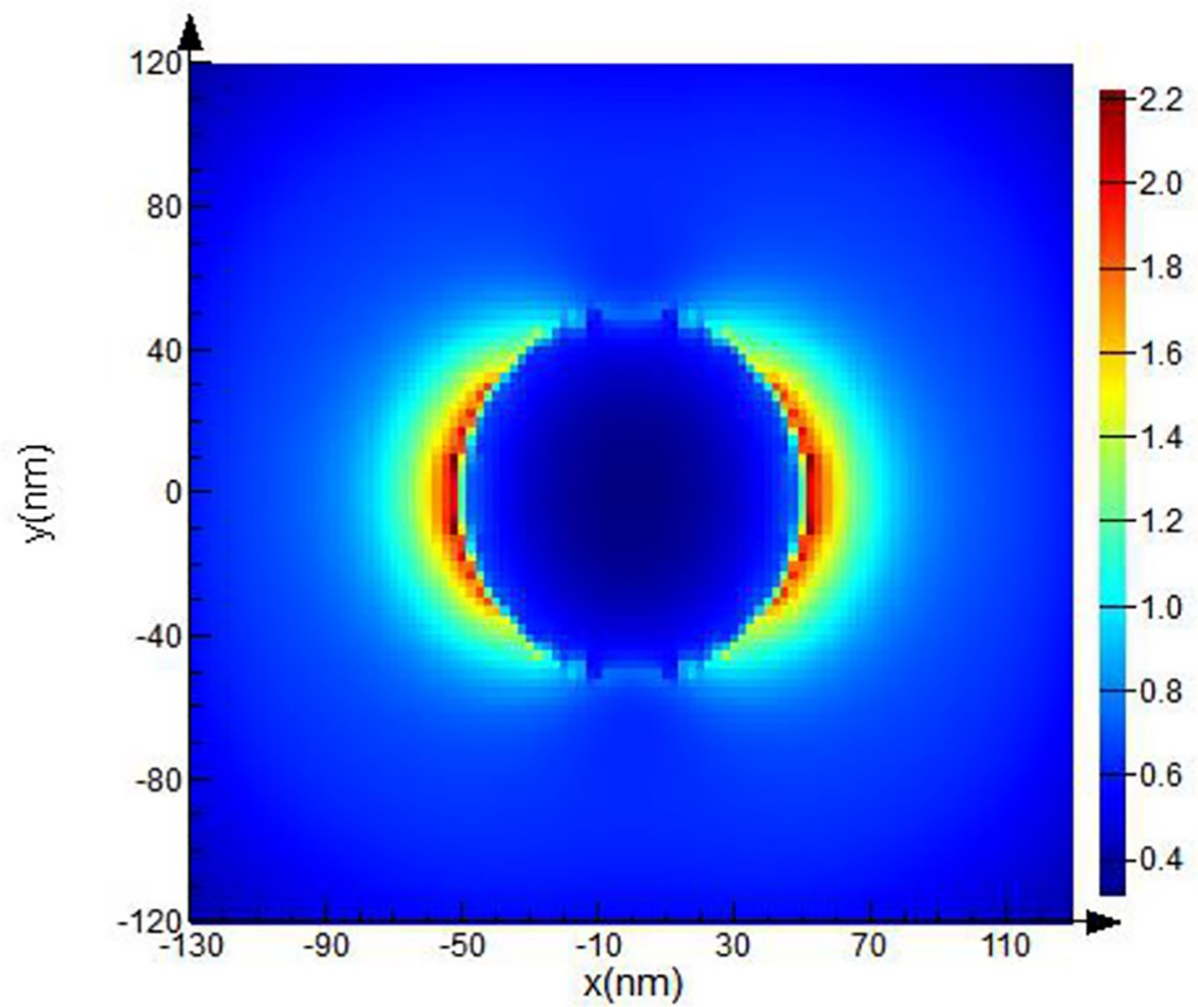
FDTD model of Pt NPs with 50 nm radii for λ = 251 nm excitation wavelength.

**Fig 4:**
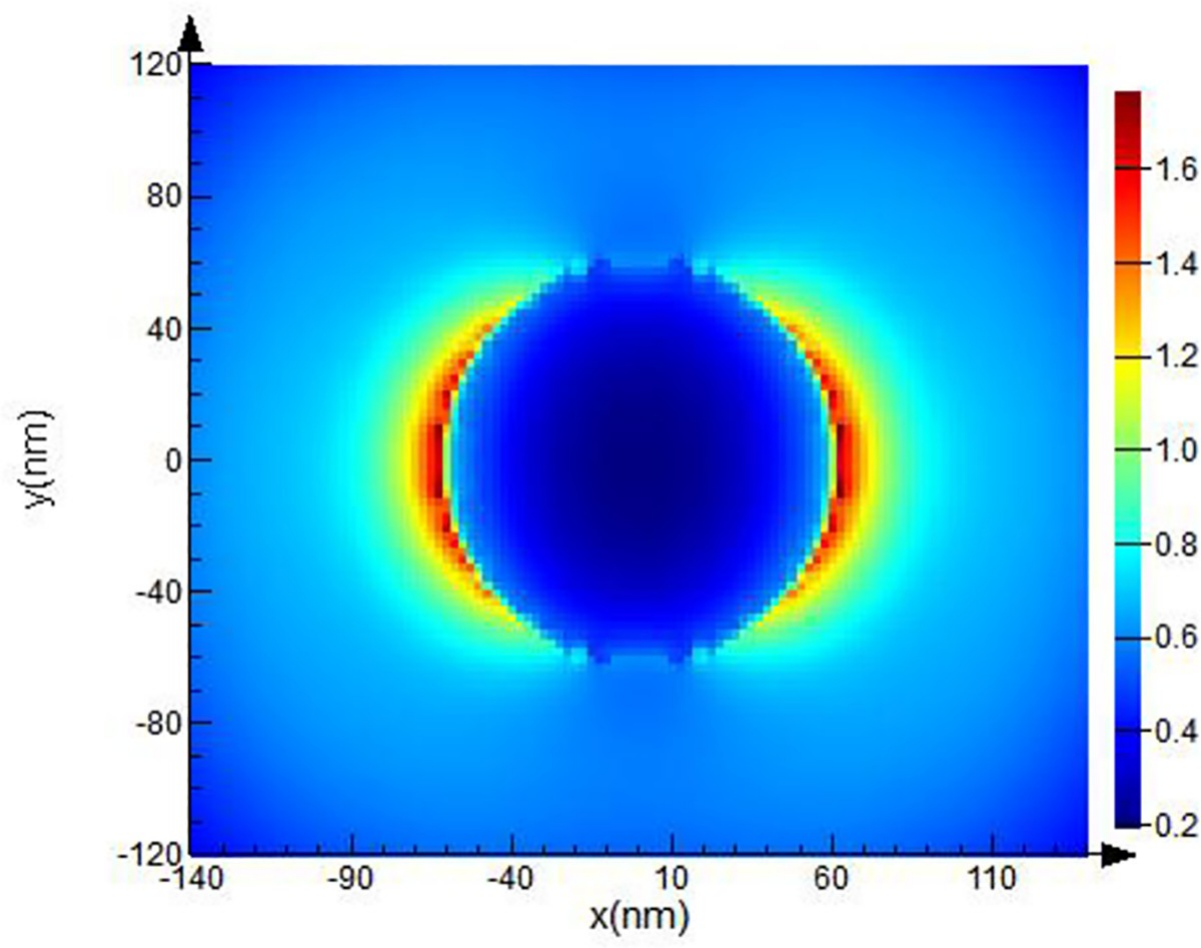
FDTD model of Pt NPs with 50 nm radii for λ = 251 nm excitation wavelength.

The Finite Difference Time Domain (FDTD)-based simulation was performed to calculate the Pt NPs optical properties and evaluate the size of NPs with maximum **E** enhancement for further NPs synthesis. We used spherical NPs morphology to perform modelling. The modeling was carried out using the Lumerical FDTD Solutions software package (v.8.19.1584, Lumerical Inc.). The modeling algorithm was described in detail in [30,35]. However, minor modifications have been performed to adjust it for spherical NPs modeling. The principal scheme of simulation process is described on Fig 1. The result algorithm consisted of five main steps and was performed as follows:

- The computational domain, mesh resolution, and boundary conditions were set. The domain used a rectangular grid in the Cartesian system. The modeling parameters (optical material properties and spherical form of the objects, electric and magnetic fields) were set at each point of the grid separately. Then, additional clarifying mesh to increase the resolution of modeling was added. The size of the counting area of the additional grid was set at the value of the grid step: *dx*, *dy*, *dz* = 2.5 nm.
- A spherical Pt NP with specified optical parameters was placed inside the computational domain. Next, the optical and geometric parameters of the body were set. We created a spherical Pt NPs with 40 nm (Fig 2), 50 nm (Fig 3), 60 nm radius (Fig 4).
- The parameters for the radiation source were set next. A total field/scattered field (TFSF) source was used and a plane wave was used to study scattering of small NPs. The TFSF source divided the computational area into two separate areas: (1) the general field area, which included the sum of the incident field wave plus the scattered field and (2) the scattered field area, which included only the scattered field. The TFSF source was used as an extended source. The physical field was the total field, and the separation into the incident and scattered fields was required a precise interpretation. For NPs in a homogeneous medium, we used *p-*polarized plane wave in our simulations. Standard simulation parameters were set as follows: the travel time of a plane-polarized wave through the working area was 1000 fs and the temperature of 300°K.
- In order to provide us with the final information about **E** distortion, the plane of the monitor was set. We used XY monitor in the frequency domain, which allowed us to collect a field profile in this domain and provide simulation results in a certain spatial domain for the FDTD solver. The monitor plane was parallel to the plane of the XY model object and perpedicular with *p*-polarized wave direction. The **E** values obtained on the XY surface. The **E** distribution can be recalculated into (EF) values in these parallel planes in a small region (~26 nm) where **E** reaches its maximum, to find the maximum possible values of EF. The energy parameters based on **E** were collected from the monitor data.
- As a last step, we calculated values of the **E**-field in our simulation. Under proper conditions, localized surface plasmons (LSP) and surface plasmon polaritons (SPP) can be revealed near the spherical Pt NPs. As described above, we used the TFSF source to simulate the electric field on a rough surface and find the maximum possible EF and intensity. Maximum EF has been detected for NPs with 40 and 50 nm radii as illustrated on Figs 2 and 3 respectively.

### Femtosecond laser ablation of Pt NPs

On the second stage, Pt NPs were fabricated by means of femtosecond laser ablation method. The 1 × 1 cm platinum metal plate (99,99% purity) was placed in the quartz cuvette with ultrapure water (Millipore Direct 3-UV, Merck) and Avesta TETA-25/30 femtosecond laser system (LTD «Avesta», Russia) unit with a TETA compressor (TETA Yb amplifier system) was used for ablation. The energy and pulse duration of the laser beam at λ = 1032 nm was 15 μJ and 280 fs, respectively. The repetition frequency and the number of pulses were set by external generator. The solution volume in cuvette was 1.5 ml, ablation time was varied from 1 to 7 minutes. The total volume for each sample was 4.5 ml. The thickness of distilled water layer over the surface metal plate was 1 mm. Each package of laser pulses was focused to a new location of the plate. To perform the ablation, the cuvette was mounted on a precise motorized positioner 8 MTF-102LS05 (Standa, Lithuania) controlled by XILab software. After ablation, the solution assumed slightly gray color.

**Fig 5:**
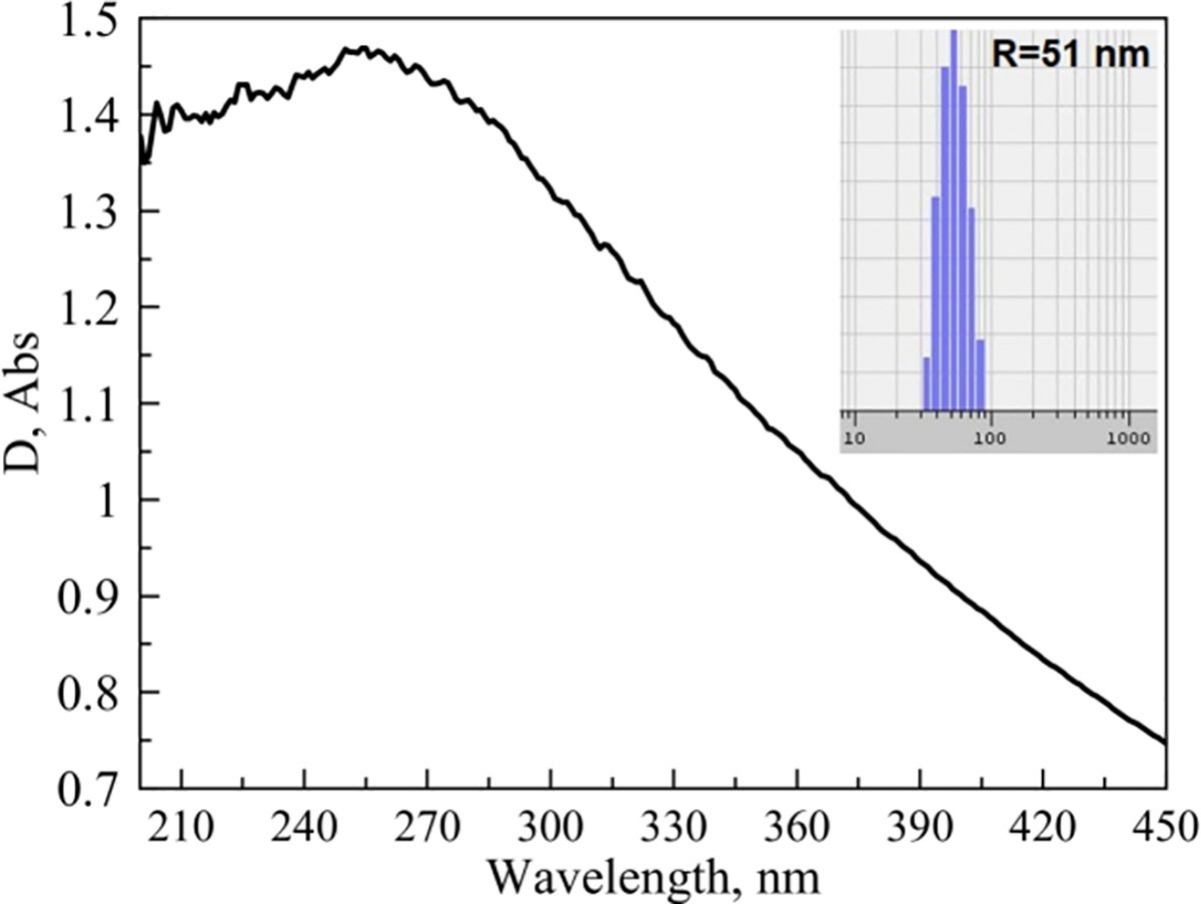
Pt NPs plasmon absorption band and NPs size distribution (inset).

**Fig 6:**
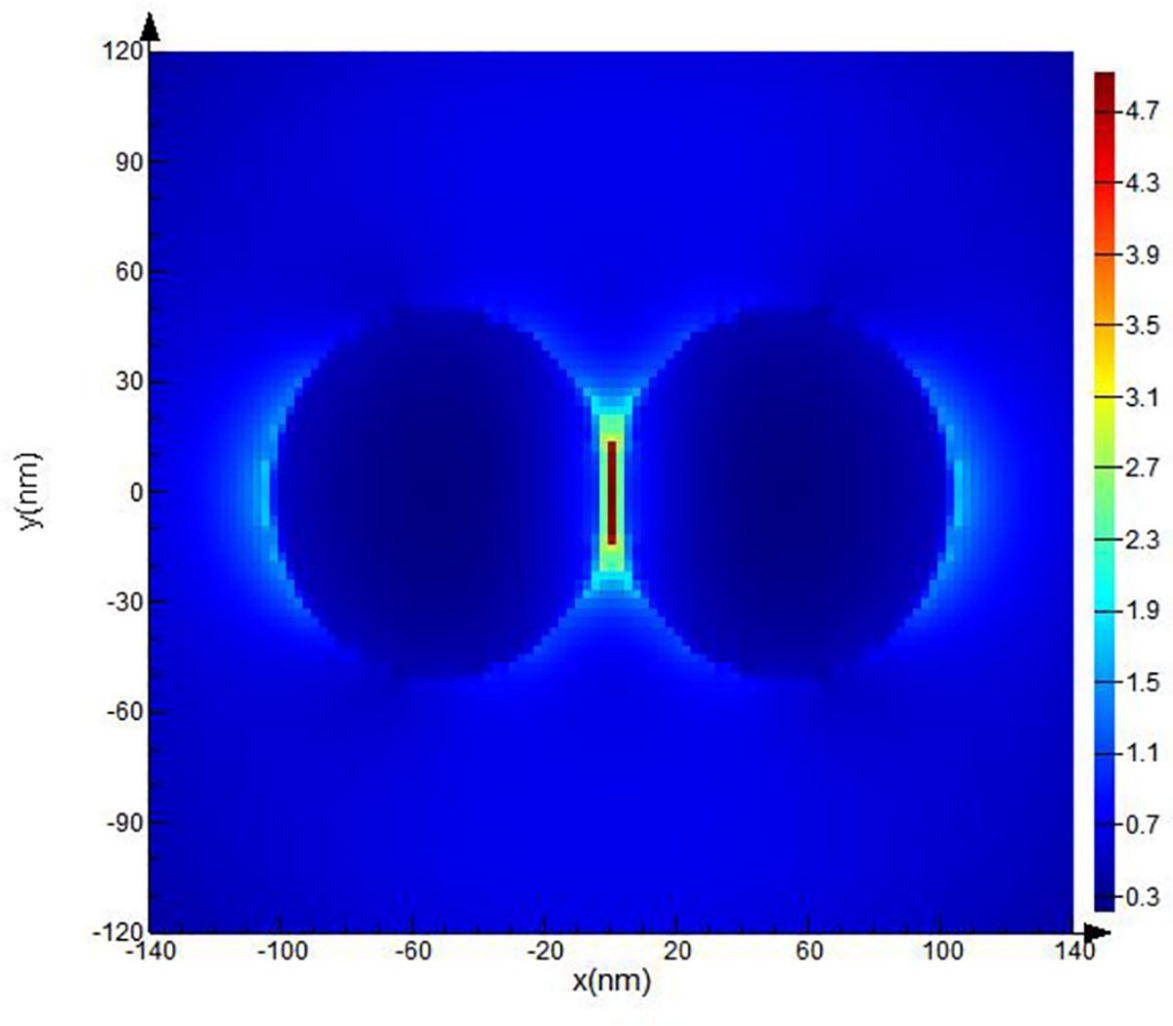
FDTD Pt NPs dimer simulation results.

The hydrodynamic radius of the obtained NPs was measured by dynamic scattering light method with PhotoCorr Complex unit (LTD «PhotoCorr», Russia) and appeared to be in range of 40 ÷ 60 nm. A Pt plasmon absorption band with a peak at λ = 251 nm has been detected using UV-2600 (Shimadzu, Japan) spectrophotometer (Fig 5). In total, 17 samples were prepared and one sample with stable plasmon absorption and radius of 51 nm (Fig 5, inset). The concentration of Pt NPs was 6.9 ⸱ 10^10^ and molar concentration was 2.5⸱10 nM. Since NPs can form aggregations, chains [36], we additionally performed Pt NPs dimer simulations illustrated on Fig 6. The maximum EF for **E** was equal 4.9.

### Platelets preparation procedure

#### Subject

15 experimental samples were taken from healthy volunteer during two-week period on 1, 4, 7, 10, and 14 days. Written informed consent had been obtained before any study procedures. All study documents including informed consent and protocol were approved by Immanuel Kant Baltic Federal University Independence Local Ethic Committee (Protocol № 8, 16.05.2019). Healthy volunteer included in the study was 35 years old and had no acute and chronic diseases. Involved patient did not take any antithrombotic drugs during the study.

#### Platelets extraction

We used preparation protocol previously described in [31] briefly, fresh venous blood samples were taken from healthy volunteer in vacuum tube containing EDTA (BD Vacutainer® spray-coated K2EDTA Tubes). It was centrifuged at 60 g for 15 min to separate platelet-rich plasma (PRP), and then the PRP was collected and placed to the new tube. Red blood cells were removed. Platelets were finally collected by further centrifugation of the supernatant at 1500 g for 15 min. All the centrifugations were carried out at 4°C using Eppendorf 5702R centrifuge. After platelet preparation, the samples were immediately taken to be examined by fluorescence spectroscopy.

#### Spectrofluorometry experiments

In order to perform fluorescence, lifetime and quantum yield measurements, 15 original PRP samples were prepared. To obtain experimental samples without and with platinum NPs, the samples were divided into two test tubes, 15 μl each. Then, to obtain the same concentration of platelets, 15 μl of an aqueous solution of sodium chloride was added to the first tube, and 15 μl of a colloidal solution of platinum NPs was added to the second tube. After that, each sample was slightly shaken. As a result, for the experimental part, 30 samples were prepared (15 samples without platinum NPs and 15 samples with platinum NPs). We used UV-VIS transparent quartz cuvettes and substrates for all spectral and time-resolved experiments. UV-VIS transparent quartz glass with a size of 4 × 4 × 1 mm was placed in 30 × 20 × 3 mm silica holder. All quartz cuvettes and holders was cleaned with isopropyl alcohol, washed with Milli-Q water (18.2 MΩ/cm) and dried at room temperature in a closed clean box. Then, 5 μl of the sample was applied to each of the two substrates and left alone until complete drying (~30 min).

#### Steady state fluorescence spectroscopy and quantum yield

The spectra of platelet mass were recorded using a Fluorolog-3 FL3-22 scientific unit (Horiba Jobin Yvon). The substrates with the platelet mass were fixed on the quartz glass and placed to the model 1933 solid specimen holder at an angle of 60° to the exciting beam. The source of continuous excitation was a 450 W xenon short arc lamp. The width of the entrance and exit slits was 5 nm. The 1200 gr/mm grating was used. Studies of the fluorescence of platelet mass were carried out in UV-range with an excitation peak of 280 nm corresponding with absorption and fluorescence spectra of the amino acid groups (Trp, Tyr, Phe), which were the part of the composition/structure of platelets. It should also be noted that the ablative Pt NPs have an absorption maximum at a wavelength of λ = 260 nm. The overlap between the absorption spectra of amino acids and Pt NPs makes it possible to observe plasmon-induced resonant energy transfer (PIRET). The quantum yield measurement was performed using an integral sphere (Quanta-φ, Horiba). The emission quantum yields of the solid samples were obtained by means of a 152 mm diameter “Quanta-φ” integrating sphere, coated with Spectralon®, using as excitation source the 450 W xenon lamp coupled with a double-grating monochromator for selecting wavelengths. Trp, Tyr and Phe (Sigma) fluorescence spectra were registered as control samples also. Gauss modeling for platelet spectra was performed using Magic Plot software.

#### Time-resolved fluorescence spectroscopy

The platelet fluorescence lifetimes were measured using Fluorolog-3 FL3-22 setup using the Data Station software. NanoLED operating at λ = 280 nm (Horiba Jobin Yvon) was used as an excitation source. The glasses with the platelet mass were fixed in the model 1933 solid specimen holder at an angle of 60° to the exciting beam. The time-to-amplitude converter (TAC) value was 100 ns (0.01455 ns/channel). Coaxial Delay was 5 nm. Slit was set as 8 nm. Peak Preset value was 1000 counts. Light emission detection was set at 330 nm. The fluorescence lifetime was calculated using the DAS6 program. A single exponential decay model was used to calculate the lifetime of fluorescence. In the calculations, the values of the standard deviation (XSQ) were within 1.15 ÷ 1.23 range.

## Results and discussion

**Fig 7:**
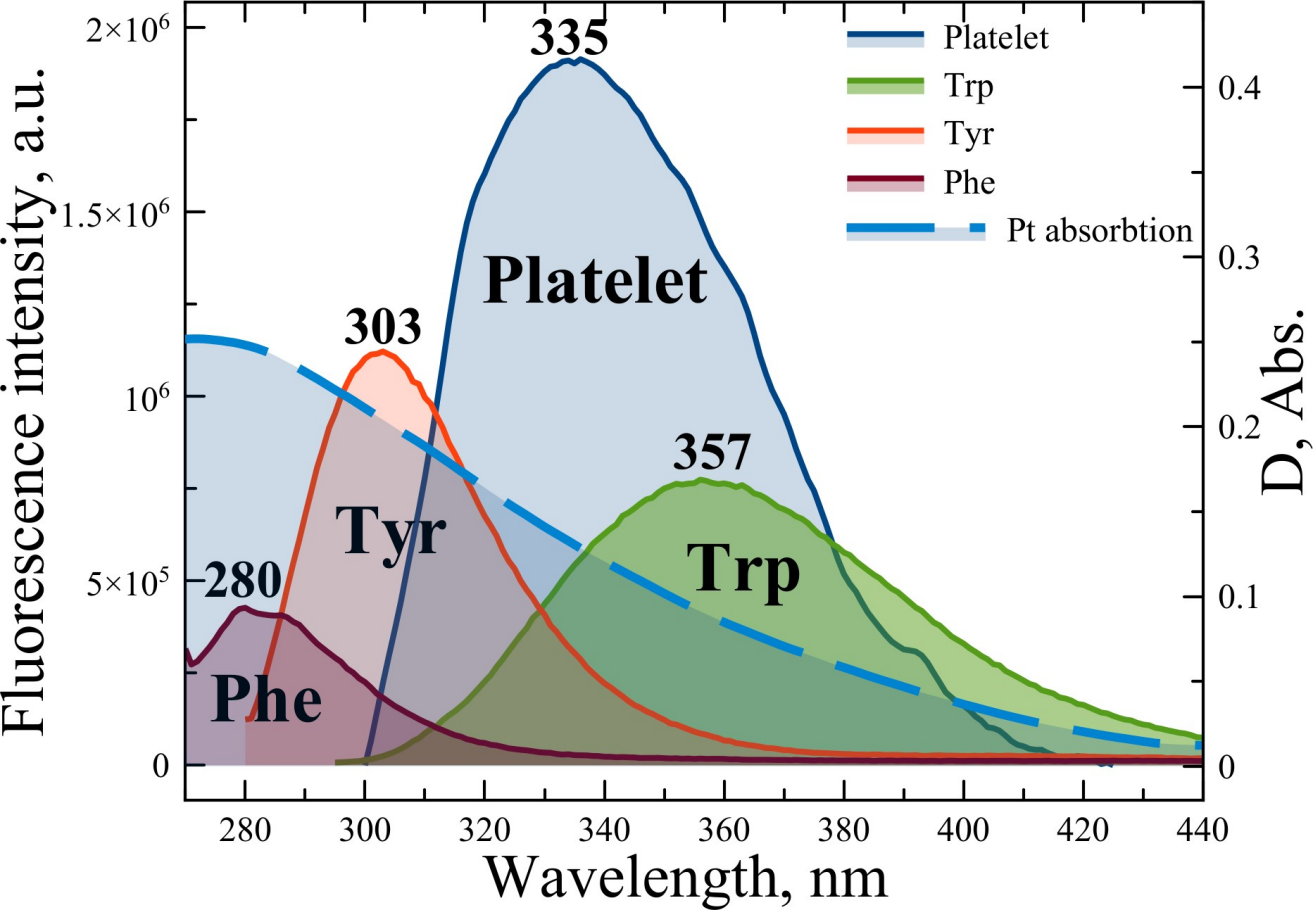
Absorption spectra of Pt NPs (blue dashed line), platelets (blue solid line), Trp (green solid line), Tyr (red solid line) and Phe (purple solid line) fluorescence spectra.

The main purpose of the article was to reveal the possibility of plasmon energy transfer between platinum NPs, the platelet membrane and its constituents. Both experimental and theoretical approaches were applied to investigate described photoprocesses. Fig 7 demonstrates fluorescence spectra of platelet and the fluorescence amino acids (controls). It can be seen from the figure that the fluorescence spectrum of the platelet with a peak at 335 nm has the maximum, partial and minimum overlap with Trp, Tyr, and Phe fluorescence spectra, respectively. From the other hand, all above-mentioned fluorescence spectra overlap with Pt plasmon resonance spectra.

**Fig 8:**
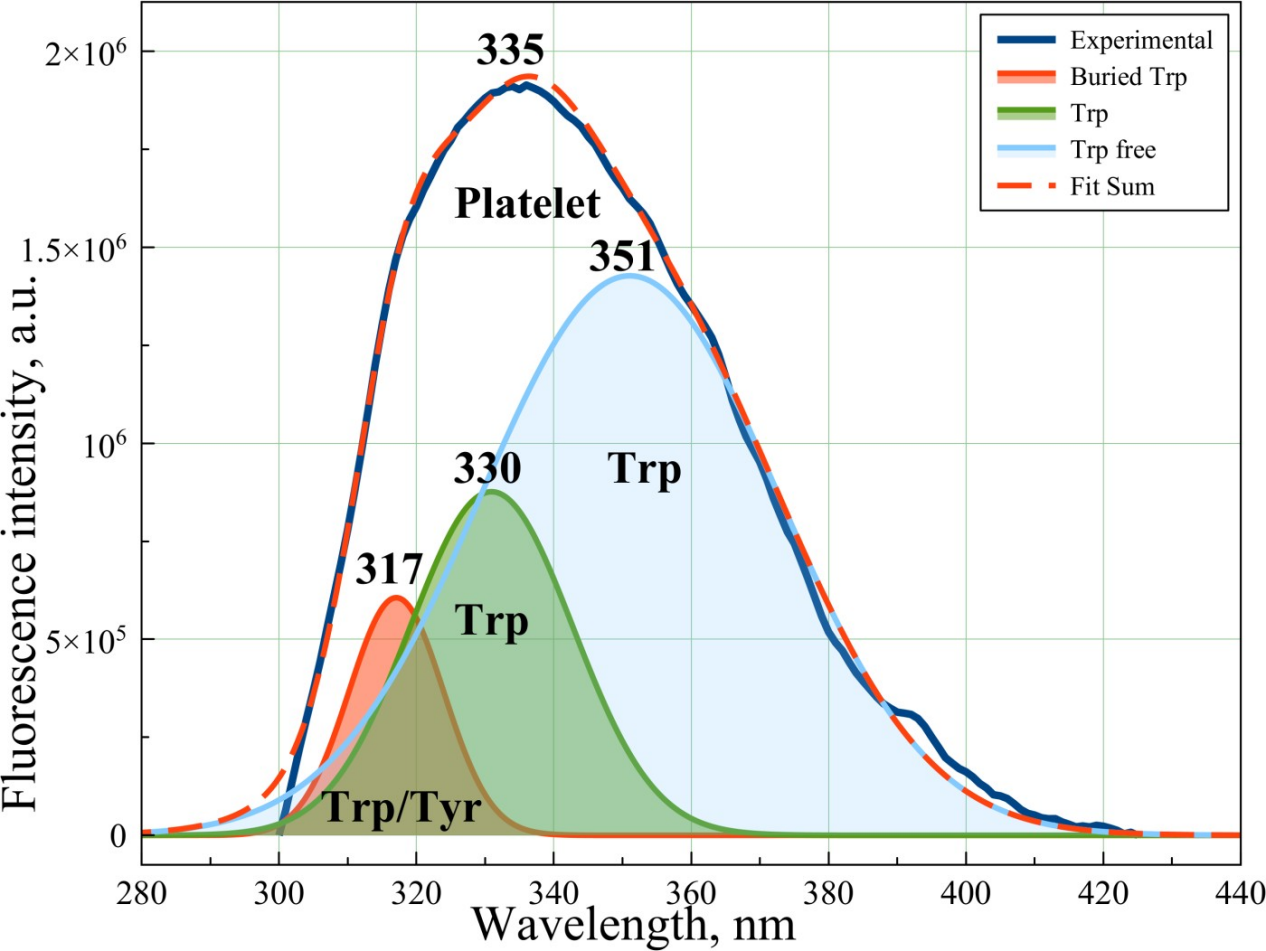
Results of Gauss modeling for platelet experimental fluorescence curve (dark blue line) including: Free Trp curve (blue line), Trp on the platelet membrane (green line) and Trp buried/masked Tyr residue (red solid line).

In this case, FRET-based models can be applied for energy transfer calculations [37]. To compare the experimental data with theory, Gauss modeling has been performed. It was shown that the spectrum could be successively modeled by three Gaussian curves (Fig 8). Comparing the theoretical and experimental data it was established that Trp peaks showed the best correlation while Tyr and Phe showed minimum and no correlation, respectively. Discussing the Trp, we supposed three Trp residues and/or one Tyr residue in our spectrum. The Trp fluorescence peak at λ = 357 nm denotes to isolated Trp in water [38]. The peak at λ = 317 nm denotes to deeply buried Trp in membrane proteins or platelet membrane or masked Tyr. The peak occurring at λ = 330 nm can denote Trp residues on the surfaces on the platelet membrane or surfaces platelet receptors [39]. It should be noted that Phe had extremely low QE [40] when excited at λ = 240 nm. The authors believe that a detailed study of this amino acid can be the object of a separate study, possibly with chromatographic isolation of this amino acid from the platelet membrane.

**Fig 9:**
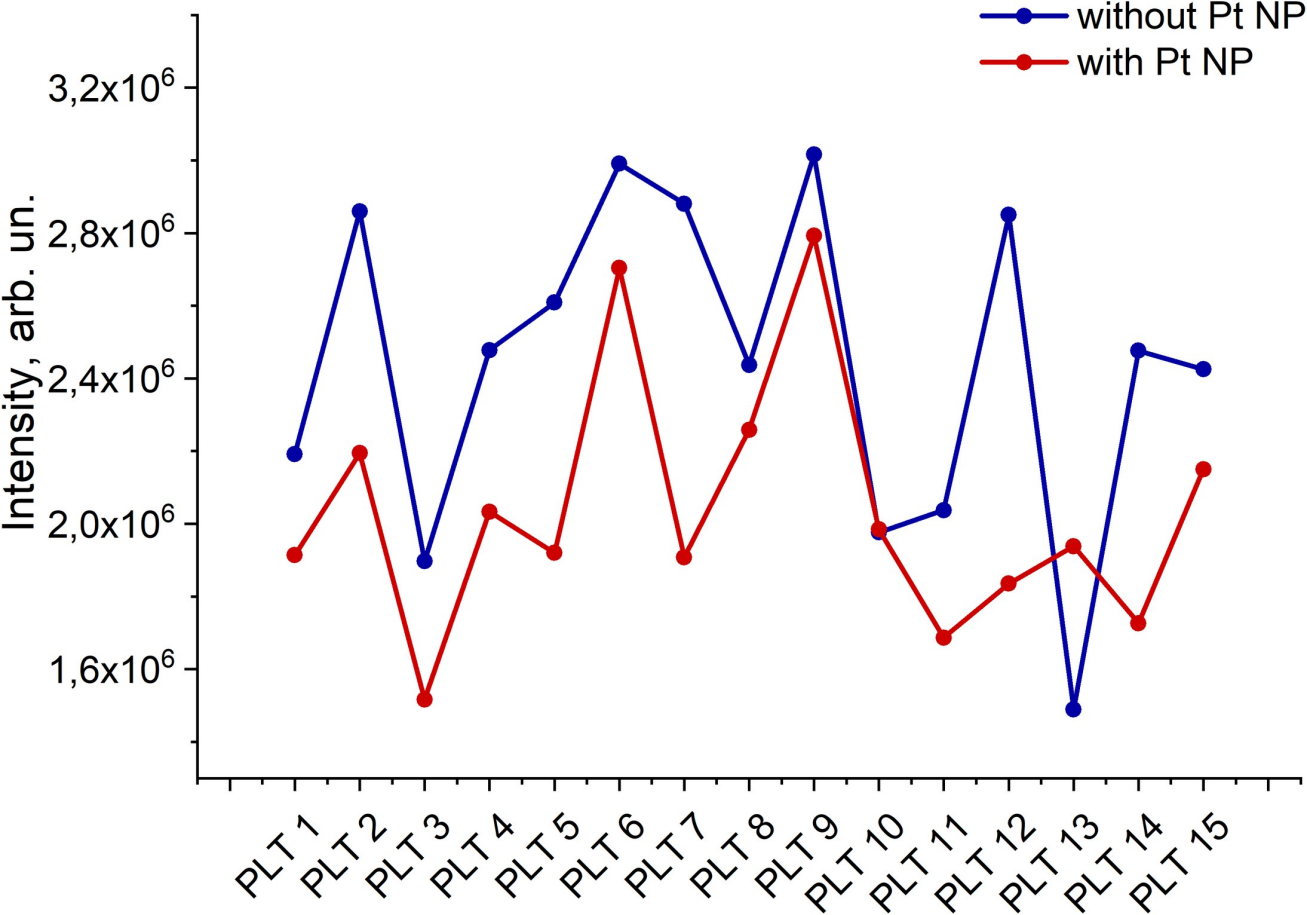
Fluorescence intensity of platelets without (blue line) and with (red line) Pt NPs.

**Fig 10:**
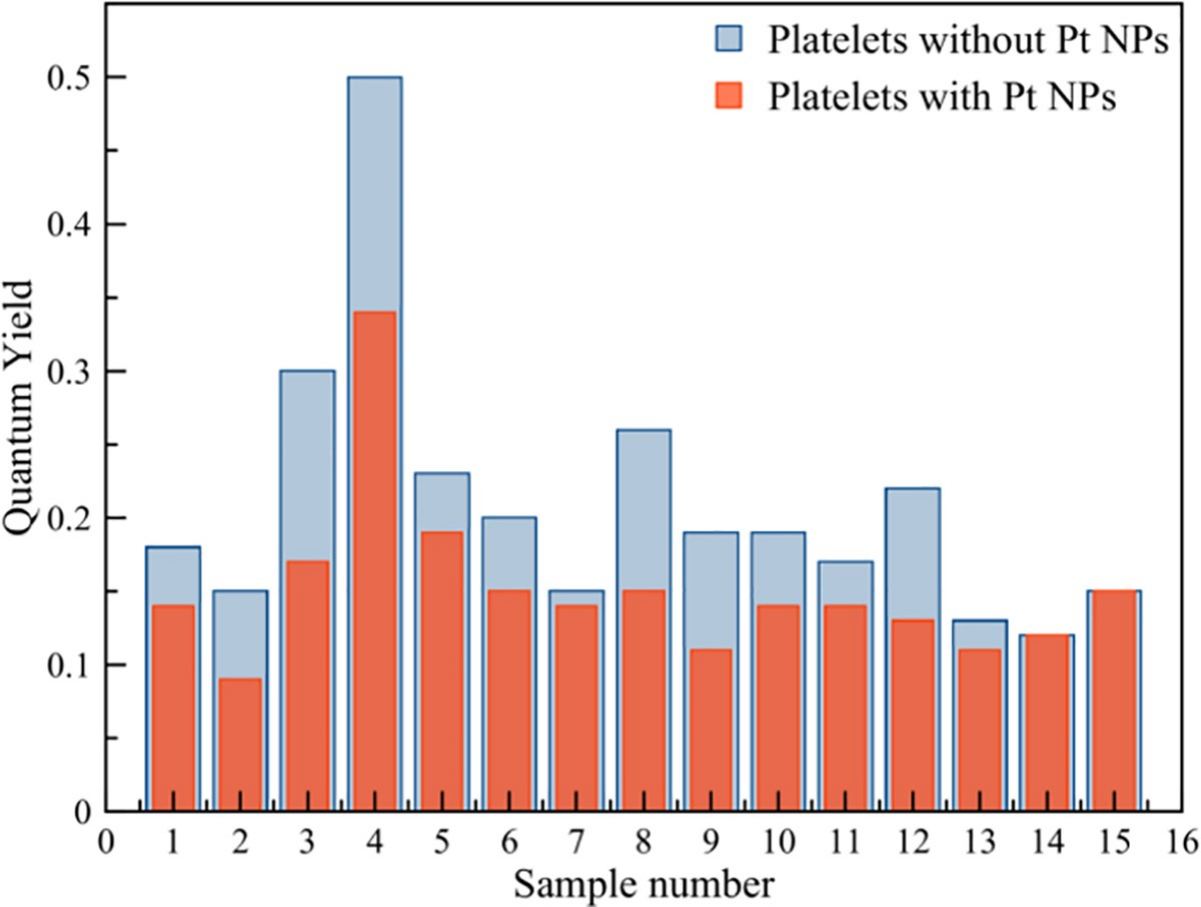
Quantum yield experimental data for platelet samples without Pt NPs (blue columns) and with Pt NPs (red columns).

According to the results of spectral measurements, fluctuations in platelet intensity were noted both in the absence of Pt NPs and in the presence of them (Fig 9). The total intensity decreased upon doping of the platelet mass with Pt NPs. In the absence of Pt NPs, the maximum fluorescence intensity reached the maximum and minimum values of 3.01 · 10^6^ and 1.43 · 10^6^ a.u, respectively. When Pt NPs doping, a smaller spread in the fluorescence intensity was observed with corresponding maximum and minimum values of 1.78 ⸱ 10^6^ and 1.41 ⸱ 10^6^. The platelet quantum yield values varied sufficiently, reaching a maximum value of 50% and a minimum value of 12%. A sufficient spread of values from sample to sample, which entails a decrease in the quantum yield, can be associated with the energy transfer between Trp residues in solution and on the membrane surface. [41]. Quantum yield values and platelet fluorescence quenching decreased from a maximum 34% for sample PLT4 to a minimum 12% for sample PLT14 (Table 1, Fig 10).

**Table 1:** Pt NPs ablation parameters.

Sample number	Pt plate mass before ablation, *g*	Pt plate mass after ablation, *g*	Ablation time, *min*	Power on sample, *mW*	Current, *A*	Mass defect, *g*	NPs concentration, *N* = Δ*m*/*m*, 10^11^
1	1.5461	1.5456	2	296	4.6	0.0005	5.9
2	1.5456	1.5446	7	296	4.6	0,001	9.4
3	1.5412	1.5408	2	408	4.9	0.0004	3.4
4	1.5408	1.5397	7	408	4.9	0.0011	3.2
5	1.5356	1.5355	2	123	4	0.0001	0.83
6	1.5330	1.5328	7	200	4.3	0.0002	1.6
7	1.5328	1.5327	2	200	4.3	0.0001	1.03
8	1.5327	1.5321	2	530	5.2	0.0006	5.1
9	1.5321	1.5309	7	530	5.2	0.0012	1.2
10	1.5264	1.5247	2	1031	6.3	0.0017	2.98
11	1.5247	1.5239	1	1031	6.3	0.0008	7.2
12	1.5177	1.5164	1	1900	8.3	0.0013	9.8
13	1.5135	1.5125	1	1383	7.3	0.001	1.1
14	1.5125	1.5123	1	555	5.3	0.0002	2.4
15	1.5123	1.5122	1	193	4.3	0.0001	1.2
**16** *	**1.4797**	**1.4791**	**2**	**230**	**4.9**	**0.0006**	**0.69**
17	1.4746	1.4740	2	270	5	0.0006	0.59

*(Used for experiment).

**Fig 11:**
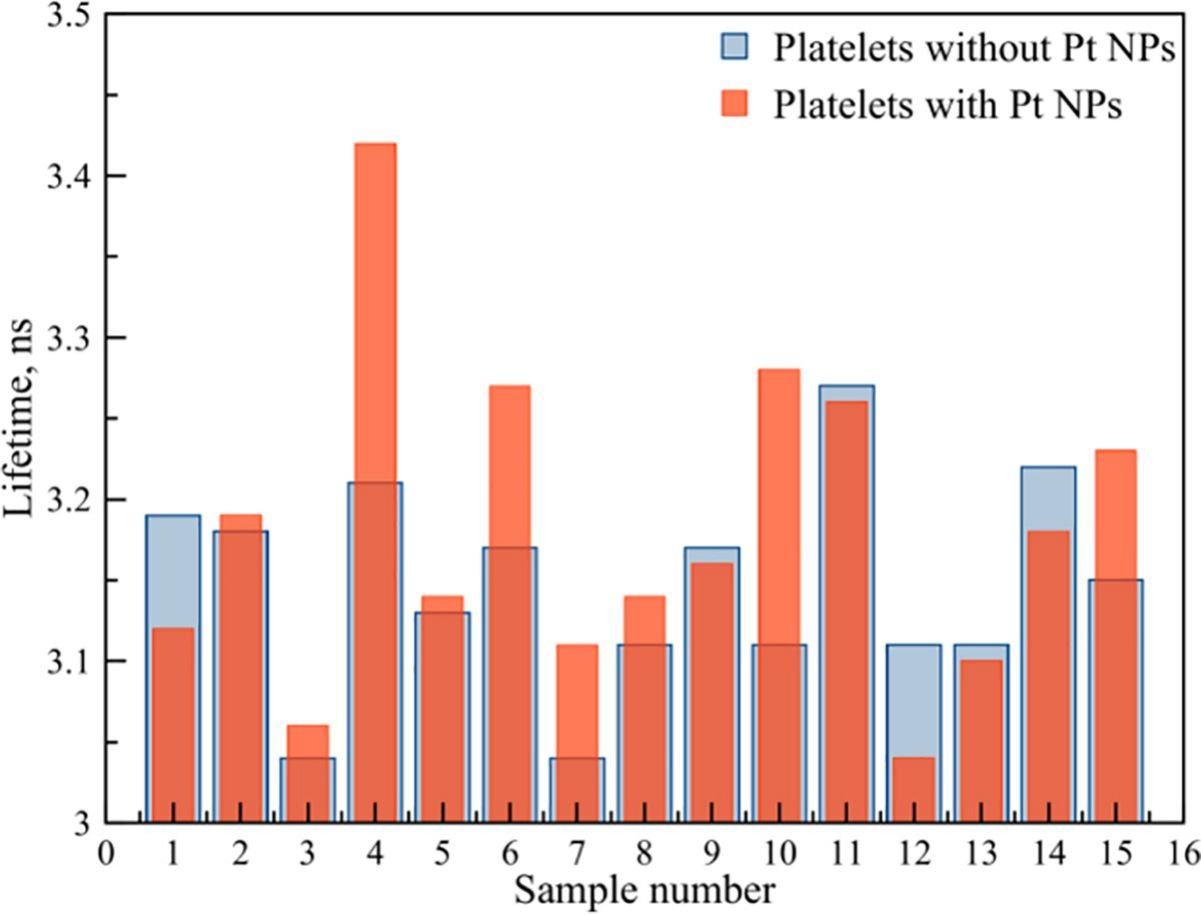
Fluorescence lifetime experimental data for platelet samples without Pt NPs (blue columns) and with Pt NPs (red columns).

Changes in the lifetime did not demonstrated significant changes both with doping of platelets with Pt NPs, and without them (Fig 11, Table 2). With Pt NPs presence, the lifetime reached its maximum value of 3.42 ns for the PLT4 sample, and the minimum one for PLT12: 3.04 ns. In the absence of Pt NPs, the maximum lifetime value (3.27 ns) was observed for sample PLT11, and the minimum (3.04 ns) for the sample 4. For the samples PLT1, PLT12, PLT13, PLT14, a decrease in the lifetimes of the excited state was detected, while for the remaining samples, their increase occurred. The platelet membrane contains all types of fluorescent amino acids and the detectable fluorescence lifetime is their joint contribution, which is also supplemented by free Trp in the solution. In this regard, the authors suggested various changes in the lifetime of amino acids that contribute to the total lifetime of fluorescence. The amount of Pt NPs in contact with the platelet membrane and individual amino acids in the platelet suspension. Also, the authors assumed the presence of dimers [42], which lead to additional changes in the photophysical parameters of the system. Previously performed FDTD simulations have demonstrated the presence of a higher amplification of **E** in dimers (Fig 6).

**Table 2:** Platelets fluorescence lifetime and quantum yield experimental data.

Sample number		Quantum yield (*φ*)	Lifetime *τ* (ns)
PLT 1	*	0.18 ± 0.0085	3.19
	**	0.14 ± 0.0103	3.12
PLT 2	*	0.15 ± 0,0087	3.18
	**	0.09 ± 0.0147	3.19
PLT 3	*	0.30 ± 0.0063	3.04
	**	0.17 ± 0.0111	3.06
PLT 4	*	0.50 ± 0.0041	3.21
	**	0.34 ± 0.0055	3.42
PLT 5	*	0.23 ± 0.0097	3.13
	**	0.19 ± 0.0081	3.14
PLT 6	*	0.20 ± 0.0055	3.17
	**	0.15 ± 0.0075	3.27
PLT 7	*	0.15 ± 0.0093	3.04
	**	0.14 ± 0.0099	3.11
PLT 8	*	0.26 ± 0.0063	3.11
	**	0.15 ± 0.0094	3.14
PLT 9	*	0.19 ± 0.0063	3.17
	**	0.11 ± 0.0125	3.16
PLT 10	*	0.19 ± 0.0075	3.11
	**	0.14 ± 0.0086	3.28
PLT 11	*	0.17 ± 0.0068	3.27
	**	0.14 ± 0.0088	3.26
PLT 12	*	0.22 ± 0.0056	3.11
	**	0.13 ± 0.0082	3.04
PLT 13	*	0.13 ± 0.0089	3.11
	**	0.11 ± 0.0101	3.10
PLT 14	*	0.12 ± 0.0097	3.22
	**	0.12 ± 0.0087	3.18
PLT 15	*	0.15 ± 0.0076	3.15
	**	0.15 ± 0.0064	3.23

*-without NPs.

**-with NPs.

We have chosen the FRET-based model to describe the energy transfer parameter due to the obvious spectral overlapping between platelet and its amino acids (donor) and Pt NPs (acceptor) (Fig 7). According to the FRET theory, the efficiency of energy transfer can be described as [43]:

$$E=1-\frac{I_{fl}}{I_{fl0}}=\frac{R_{0}^{6}}{R_{0}^{6}+r^{6}}=1-\frac{\tau_{DA}}{\tau_{D}}$$ (1)

where *I*_*fl*_ and *I*_*fl*0_ denote fluorescence intensity in the presence and absence of NPs, respectively, *r* is distance between acceptor and donor, *R*_0_ denotes Förster radius at which the efficiency is 50%, τ_*DA*_ is the donor lifetime in the presence of an acceptor, τ_*D*_ is the donor lifetime in the absence of an acceptor. *R*_*0*_ can be calculated using the formula:

$$R_{0}=0.2108{\left\{\frac{k^{2}\varphi }{n^{4}}\int_{0}^{\infty }I_{D}(\lambda )\xi_{A}(\lambda )\lambda^{4}d\lambda \right\}}^{\frac{1}{6}}$$ (2)

где *k*^2^ denotes orientation factor, for liquid media making up the value equal 1/3, *n* is medium refractive index, φ denotes donor fluorescence quantum yield (*D*), *I*_*D*_(λ) denotes donor fluorescence intensity at wavelength λ, ξ_*A*_(λ) is the acceptor molar absorption coefficient (*A*).

The rate of FRET was calculated using the ratio:

$$k_{dd}=\frac{1}{t_{d}}{\left(\frac{R_{0}}{r}\right)}^{6}$$ (3)

The distance *r* between the donor and acceptor was calculated by the formula:

$$r=R_{0}{\left(\frac{1}{E}-1\right)}^{\frac{1}{6}}$$ (4)

The data obtained as a result of calculations are shown in Table 3.

**Table 3:** Calculated FRET parameters.

Sample number	Overlap integral (*J*), 10^17^ nm^4^/(M⸱cm)	Förster radius *R*_0_(A)	NP–platelet distance r, A	K_dd,_ 10^8^ s^-1^	Energy transfer efficiency E
PLT 1	5.17	38.8	65.5	1.67	0.02
PLT 2	5.17	38.8	77.1	1.06	0.01
PLT 3	5.16	38.7	66.2	1.91	0.04
PLT 4	5.17	38.8	58,6	1.68	0.06
PLT 5	5.16	38.7	77.1	0.69	0.01
PLT 6	5.17	38.8	77.1	2.19	0.03
PLT 7	5.16	38.7	65.1	2.00	0.02
PLT 8	5.16	38.7	69.3	0.61	0.01
PLT 9	5.20	39.0	77.1	0.84	0.01
PLT 10	5.17	38.8	77.9	3.72	0.05
PLT 11	5.14	38.5	77.6	0.94	0.01
PLT 12	5.15	38.6	60.1	1.37	0.02
PLT 13	5.14	38.5	76.7	1.23	0.01
PLT 14	5.14	38.5	69.2	1.33	0.01
PLT 15	5.15	38.6	76.7	2.92	0.03

According to the results of calculations, it was found that *R*_0_ does not undergo significant changes and is equal to 38 nm, while the distance between Pt NPs and platelet fluctuates in the 65 ÷ 78 nm range. Despite the fact that the transfer efficiency was rather low (1 ÷ 6%), the rate of dipole-dipole transfer was high, reaching 1.91⸱10^8^ s^-1^ for the sample PLT3. The high rate of energy transfer can be explained because of the small distance between platelet and NP. This result correlates with energy transfer mechanisms and it explanations described in [44,45]. Fluorescence, quantum yield and platelet amino acid lifetime changes in presence and without femtosecond ablated platinum NPs have been studied. Förster resonance energy transfer model was applied for calculations of energy parameters. The possibility of energy transfer between Pt NPs and platelet proteins have been revealed. The significant prospects of using Pt NPs as a basis for quenching sensor have been demonstrated. Pt NPs based sensors can be used for platelet membrane and it receptors investigation in case of antiplatelet drug therapy control, thrombus formation processes or other cardiovascular complications. It should be noted, that other amino acids, like His can be also a perspective candidate for future fluorescent study using λ = 200–250 nm excitation wavelengths [46].

## Supporting information

S1 Data (RAR)
